# On-chip mode-selective manipulation based on the modal-field redistribution assisted with subwavelength grating structures

**DOI:** 10.1515/nanoph-2023-0111

**Published:** 2023-03-22

**Authors:** Xiaolin Yi, Chenlei Li, Weike Zhao, Long Zhang, Yaocheng Shi, Daoxin Dai

**Affiliations:** State Key Laboratory for Modern Optical Instrumentation, Center for Optical & Electromagnetic Research, College of Optical Science and Engineering, International Research Center for Advanced Photonics, Zhejiang University, Zijingang Campus, Hangzhou 310058, China; Ningbo Research Institute, Zhejiang University, Ningbo 315100, China

**Keywords:** mode-selective manipulation, multimode, silicon photonics, subwavelength grating

## Abstract

Efficient mode-selective manipulation in multimode photonics has drawn much attention as a key technology for realizing scalable and flexible mode-division multiplexing (MDM) systems. A mode-selective manipulation scheme based on the modal-field redistribution assisted with subwavelength grating (SWG) structures is proposed and demonstrated for the first time. In particular, the proposed scheme focuses on manipulating the coupling coefficient *κ* as well as the ratio *δ*/*κ* for different mode channels. The SWG structures are used to engineer the refractive-index profile and redistribute the modal field distributions in the multimode bus waveguide, so that different modes are localized in different local regions. In this way, the undesired mode coupling can be suppressed significantly while the desired mode coupling can be enhanced. With such mode manipulation scheme, the fundamental and higher-order mode channels in the bus waveguide can be added/dropped independently and freely. As a proof of concept, a three-channel mode-selective add-drop coupler utilizing the proposed scheme is fabricated and demonstrated experimentally on silicon. The fabricated devices show low excess losses ranging from 0.1 to 1.9 dB over a wavelength range of 70 nm. The inter-mode crosstalks are lower than −19.4 dB in the wavelength range of 1525–1600 nm. The crosstalks for the drop and through ports (i.e., the residual power) are suppressed to be as low as −18 ∼ −30 dB in the wavelength range of ∼60 nm with the assistance of an additional coupler in cascade for performance improvement. The present concept of manipulating the evanescent coupling of the mode-channels paves the way for designing multimode silicon photonic devices with flexible mode-selective manipulation for MDM systems.

## 1 Introduction

With the unprecedented growing demand for data communication capacity, the conventional electrical interconnect technology is facing the bottleneck of the transmission capacity and the power consumption [1]. On-chip optical interconnect is regarded as a promising approach with inherent advantages of broad bandwidth, high speed and low power consumption [2–4]. The ultra-high link capacity of optical interconnects can be achieved by utilizing various types of multiplexing technologies developed in the past decades, including wavelength-division multiplexing (WDM) [5–7], polarization-division multiplexing (PDM) [8–10] and mode-division multiplexing (MDM) [11–13]. Among them, the MDM technology has emerged as a promising solution with great potential by utilizing orthogonal modes to multiplex signals within a single-wavelength carrier, which paves the way for further enhancing the link capacity of optical interconnects [14].

Currently the MDM technology is attracting more and more attention because of its excellent scalability and cost-effectiveness, and numerous smart designs of multimode photonic devices have been developed widely to be better operated with higher-order modes, including mode (de)multiplexers [15–17], multimode waveguide crossings [18–20], multimode waveguide bends [21–23] and multimode power splitters [24–26]. Many of them are developed with silicon photonics, which is considered as a remarkable platform due to its high integration density and excellent complementary metal-oxide semiconductor (CMOS) compatibility [27, 28]. For multimode silicon photonics [29], the realization of effective mode manipulation has been one of the major demands since both the fundamental mode and the higher-order modes are involved, which may lead to some trouble due to the undesired excess loss (EL) and inter-mode crosstalk. In mode (de)multiplexing scenarios, great efforts have been made to realize efficient mode manipulation in recent years, so that certain mode channels can be added/dropped selectively by using some specific structures, such as multimode interference (MMI) couplers [30–32], asymmetric Y-junctions [33, 34], asymmetric directional couplers (ADCs) [35–37], adiabatic couplers [38, 39] and inverse-designed structures [40]. In recent years, subwavelength-grating (SWG) structures have also been introduced to improve the capacity and performance of on-chip mode (de)multiplexing. These SWG structures provide high design flexibility to manipulate the effective indices of the guided modes for mode (de)multiplexers, which is helpful for achieving shortened coupling regions and broadened bandwidth [41–44]. Nevertheless, the aforementioned types of mode (de)multiplexers are still far from realizing high-flexibility mode manipulation desired for the device design.

For example, it is still difficult to selectively add/drop any one of the mode channels to/from the multimode bus waveguide (MBW). Particularly, adding/dropping the fundamental mode is a big challenge for most structures used for mode (de)multiplexing, including the ADCs which have been used very commonly. Recently, some solutions for the selective access of arbitrary mode carriers have been proposed [45, 46]. In Ref. [45], the solution is introducing an *additional* mode exchanger to convert the lower-order mode to the highest-order mode (which can be added/dropped easily). With this idea, one can access any one of the mode-channels with the help of the corresponding mode exchanger and a traditional ADC designed for coupling the highest-order mode in the MBW to the fundamental mode in the access waveguide. In Ref. [46], multimode microring resonators with ADCs are introduced to help add/drop arbitrary mode carriers, which however works wavelength-selectively and is unavailable for broadband operation. Therefore, it is still desired to find a solution for manipulating all the mode-channels in a simple and convenient way. As it is well known, for the most commonly used ADC, which consists of a *multimode* bus waveguide and a *singlemode* access waveguide, the structure is designed according to the phase matching condition between the target guided-mode in the bus waveguide and the fundamental mode in the access waveguide. In this design, people mainly focus on the optimization of the waveguide core widths to match the propagation constants of these two modes to be coupled, while little attention has been paid to the manipulation of the mode coupling coefficients in the ADC for all the guided-modes in the bus waveguide.

In this paper, we propose a novel mode-selective manipulation scheme implemented by redistributing the mode field with the assistance of SWG structure for the first time. This possibly enables the desired manipulation of the evanescent coupling for any mode-channels. As an example, here we mainly consider the three lowest-order transverse-electric modes (i.e., TE_0_, TE_1_ and TE_2_ modes). Particularly, the SWG structure is introduced to engineer the refractive index and the mode profiles in the MBW. Therefore, the launched TE_0_, TE_1_ and TE_2_ modes are redistributed to be localized in different regions appropriately and consequently one can have an enhanced coupling coefficient for the mode-channel in the MBW desired to be added/dropped. Meanwhile, the coupling coefficient of the undesired mode-channels is suppressed significantly with the help of the field redistribution, resulting in low inter-mode crosstalk. With this design, all the mode channels (including the fundamental mode) in the MBW can be efficiently added/dropped independently and freely without introducing extra devices. As a proof of concept, we present a three-channel mode-selective add-drop coupler utilizing the proposed mode manipulation scheme, which exhibits low ELs less than 0.3 dB over a wavelength range of 70 nm for all the mode channels. The inter-mode crosstalks for the TE_0_, TE_1_ and TE_2_ mode-channels are <−23.7 dB (TE_1_ input), −26.5 dB (TE_0_ input) and −31.1 dB (TE_0_ input), respectively. For the fabricated devices, all three modes have ELs of 0.1–1.9 dB over a wavelength range of 70 nm and inter-mode crosstalks lower than −19.4 dB in the wavelength range of 1525–1600 nm. The crosstalks between the drop and through port (i.e., residual power) are suppressed to be as low as −18 ∼ −30 dB in the wavelength range of ∼60 nm with the assistance of an additional coupler in cascade for performance improvement. It is expected that the proposed concept of manipulating the evanescent coupling of the mode-channels in the multimode bus waveguide can be employed to many other multimode photonics applications and further extended to various material platforms.

## 2 Principle and design

**Figure 1:**
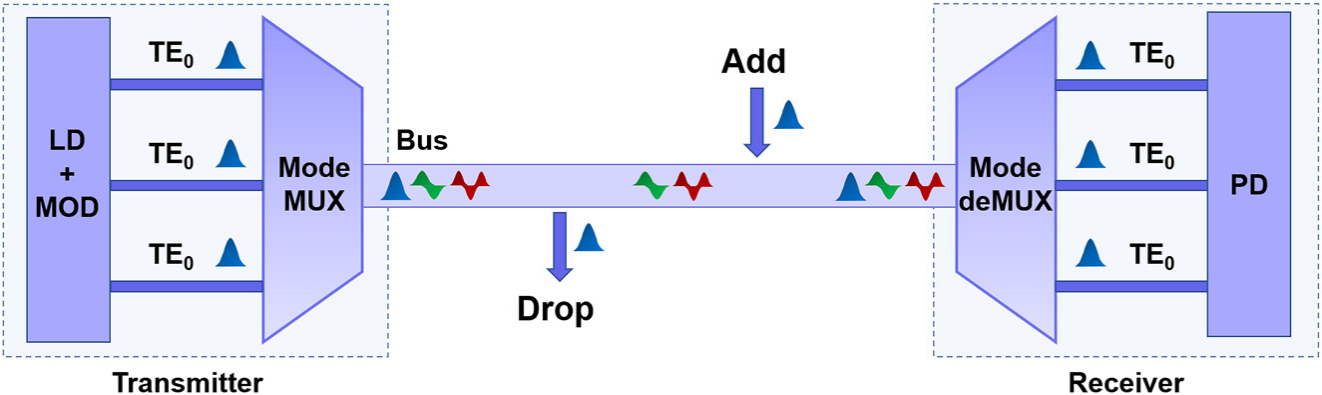
The scenario of adding/dropping any mode channels (including the fundamental one) to/from an MBW in an MDM system. LD: laser diode, MOD: modulator, Mode (de)MUX: mode (de)multiplexer, PD: photodetector.

**Figure 2:**
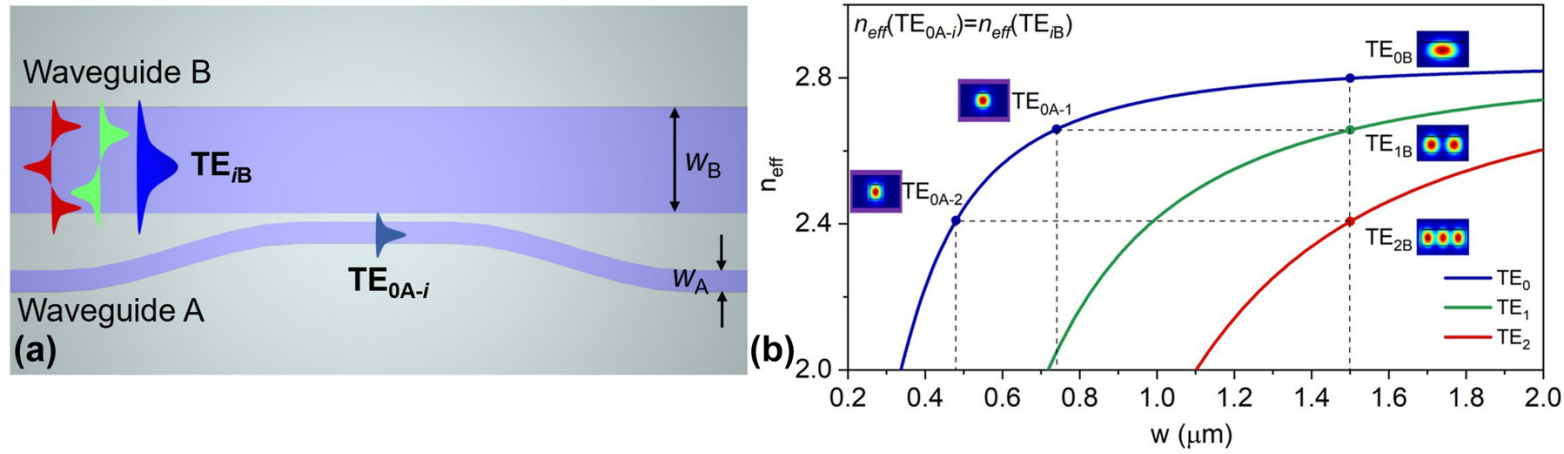
Adding/dropping mode channels with traditional ADC structure. (a) Traditional ADC used commonly for adding/dropping the mode channels (e.g., TE_0B_, TE_1B_ and TE_2B_) to/from an MBW. (b) Calculated effective indices of the guided-modes in an SOI strip waveguide as the core width varies.

In this paper, we consider the scenario of adding/dropping any mode channels to/from an MBW in an MDM system, as shown in Figure 1. As it is well known, ADCs are commonly used as basic components with high scalability for multiplexing/demultiplexing the mode channels in the transceivers of an MDM system [41, 42, 47]. In particular, a traditional ADC usually consists of an access waveguide (waveguide A) and a bus waveguide (waveguide B) with different core widths (*w*_A_ and *w*_B_), which are usually chosen according to the phase matching condition, as shown in Figure 2(a) and (b). When considering the evanescent coupling between the fundamental mode in waveguide A and the *i-*th guided-mode in waveguide B for an ADC, according to the coupled mode theory [48], the maximal coupling power ratio *F* is given by

(1) $$F=\frac{1}{1+{\left(\delta /\kappa \right)}^{2}},$$

where *κ* is the coupling coefficient and *δ* = (*β*_*i*B_ − *β*_0A_)/2. Here *β*_0A_ and *β*_*i*B_ are the propagation constants of the fundamental mode in waveguide A and the *i-*th mode in waveguide B, respectively. When designing an ADC, one usually chooses the core widths (*w*_A_ and *w*_B_) optimally to make *δ* = 0 (i.e., *β*_0A_ = *β*_*i*B_). Accordingly, one has *F* = 1, indicating that the fundamental mode in waveguide A can be coupled completely to the *i-*th mode in waveguide B as desired. Meanwhile, for the *j-*th mode in waveguide B (where *j* ≠ *i*), one has *β*_0A_ ≠ *β*_*i*B_ automatically due to the mode dispersion. Consequently, the coupling between them (the fundamental mode in waveguide A and the *j-*th mode in waveguide B) is much less than 100% if *δ*/*κ* >> 1. Figure 2(b) shows the calculated results for the effective indices of the TE_0_, TE_1_ and TE_2_ modes in a silicon-on-insulator (SOI) strip waveguide as the core width varies. It can be seen that the effective index of the fundamental mode TE_0A-*i*_ in the access waveguide can be selectively matched to that of the targeted TE_*i*B_ mode-channel (*i* = 0, 1, 2, …) in the MBW by appropriately choosing the core width *w*_A_ of the access waveguide. For example, the TE_1B_ and TE_2B_ higher-order modes in the 1.5 μm-wide MBW can be matched selectively to the fundamental mode of the access waveguide with a core width of *w*_A_ = 0.74 μm and 0.48 μm, respectively. On the other hand, the other guided-modes in the MBW becomes non-matched automatically due to the mode dispersion. As a result, mode-selective evanescent coupling is possible to be achieved for matching higher-order mode in the MBW.

As it might be noticed, there has no much attention paid on suppressing those undesired mode coupling in those works published previously. In the design of ADCs, sometimes one might try to maximize the mismatch *δ*, which helps suppress the undesired mode coupling. Unfortunately, it is hard to achieve a large *δ* for an ADC with a wide MBW which has relatively small mode dispersion. As a result, it becomes more difficult to achieve low intermode crosstalk when more mode-channels are involved in a wider MBW. Furthermore, it is hard to design an ADC enabling mode-selective coupling for the fundamental modes in the MBW and the access waveguide by simply choosing different core widths. Particularly, utilizing a DC with identical core width cannot do the work either even when one carefully manipulates the coupling lengths for different mode-channels. A potential solution is to introduce the mode conversion from the fundamental mode to the highest-order mode, as demonstrated in [45]. Alternatively, noting that the evanescent coupling in ADCs is actually determined by the ratio *δ*/*κ* (instead of *δ* itself) as indicated by Eq. (1), in this paper we focus on manipulating the coupling coefficient *κ* and the ratio *δ*/*κ* in mode-selective coupling systems, which is totally different from the ideas reported previously. Basically, it is desired to maximize the coupling ratio *κ* for the desired mode-channel and minimize the coupling coefficients *κ* for the other undesired mode-channels. Here we propose and demonstrate a novel mode-selective manipulation scheme based on the modal-field redistribution for the first time by introducing SWG structures.

**Figure 3:**
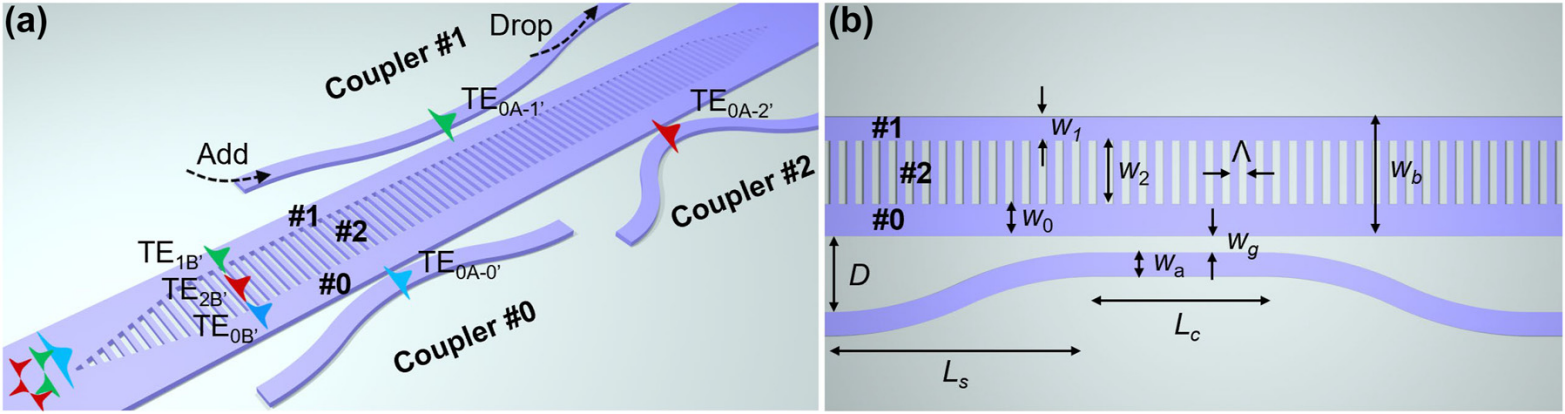
Mode-selective manipulation scheme utilizing modal-field redistribution. (a) Illustration of the proposed mode-selective manipulation scheme. (b) Top view of the SWG mode coupling region labeled with key parameters.

Figure 3(a) and (b) shows the illustration of the proposed mode-selective manipulation scheme, which enables the simultaneous manipulation of the evanescent coupling for all the mode-channels considered. The proposed structure consists of two mode-evolution regions and a mode-coupling region inserted between them. Particularly, an SWG structure is used to engineer the refractive index, so that the mode fields in the MBW are redistributed. By optimally designing the SWG mode-evolution region, the launched TE_0_, TE_1_ and TE_2_ modes in the MBW are converted efficiently to the TE_0B′_, TE_1B′_ and TE_2B′_ supermodes, which are localized at region #0, #1 and #2 in the mode-coupling region, respectively, as shown in Figure 3(a).

One might notice that the redistributed supermodes have varied effective indices, which are different from those of the originally launched modes in the MBW. In the mode-coupling region shown in Figure 3(b), the redistributed supermodes (TE_0B′_, TE_1B′_ and TE_2B′_) are then evanescently coupled to the fundamental modes in the corresponding access waveguide placed aside. The widths of the access waveguides are chosen according to the phase-matching condition. In this way, the peak of the redistributed mode field of the channel desired to be added/dropped is closed to the corresponding access waveguide, indicating that the coupling coefficient *κ* is enhanced. Therefore, the launched mode has efficient evanescent coupling with the fundamental mode in the access waveguide as desired when the core widths are chosen optimally. Meanwhile, the other modes undesired to be added/dropped are localized far away from the access waveguide, which means that the coupling coefficient *κ* is minimized significantly and thus the coupling could be suppressed well. Interestingly, in this case the mode-selective coupling for the fundamental mode in the MBW becomes available by simply optimizing the core widths and the length of the coupling region, which is of great importance to MDM systems. For the MDM system shown in Figure 1, the presented coupler can be placed at the MDM transmission link, and any modes (including the fundamental one) can be selectively added/dropped from the bus waveguide. As a summary, the proposed mode-selective manipulation scheme, which utilizes the modal field redistribution with the help of SWG structures, enables the manipulation of not only the mismatch *δ* but also the coupling coefficient *κ* for all the mode channels, which is totally different from the design of traditional ADCs. In particular, this is promising for the scenario of adding/dropping mode channels to/from an MBW and is possible to be used further for other multimode photonics applications in the future.

Here we use the SOI wafer with a 220 nm-thick top silicon layer and a 2-μm-thick buried-oxide layer. The operation wavelength is around 1550 nm, and the corresponding refractive index of silicon and silica are *n*_Si_ = 3.47 and *n*_SiO2_ = 1.444, respectively. As an example, the MBW width *w*_b_ is chosen as 1.5 μm to support no less than three mode-channels of TE polarization. Notably, the end widths of regions #0, #1 and #2 (i.e., *w*_0_, *w*_1_, *w*_2_) should be designed carefully to ensure that the redistributed supermodes are localized at the desired local regions of the MBW. The design rules for determining (*w*_0_, *w*_1_, *w*_2_) are described as follows.

(1) The widths *w*_0_, *w*_1_ and *w*_2_ should be chosen appropriately so that the supermode dispersion is maximized for lowering the intermode crosstalk. One should maximize the difference among the effective indices *n*_eff_(TE_0B′_), *n*_eff_(TE_1B′_) and *n*_eff_(TE_2B′_) of the supermodes. Basically the widths *w*_0_, *w*_1_ and *w*_2_ are chosen appropriately so that one has *n*_eff_(TE_0B′_) > *n*_eff_(TE_1B′_) > *n*_eff_(TE_2B′_).
(2) The widths *w*_0_, *w*_1_ and *w*_2_ should be sufficiently large (i.e., >0.2 μm) so that the supermodes can be supported well.
(3) The width *w*_2_ should be sufficiently large to ensure that the supermodes can be localized well in the corresponding region with only one major peak as desired.

**Figure 4:**
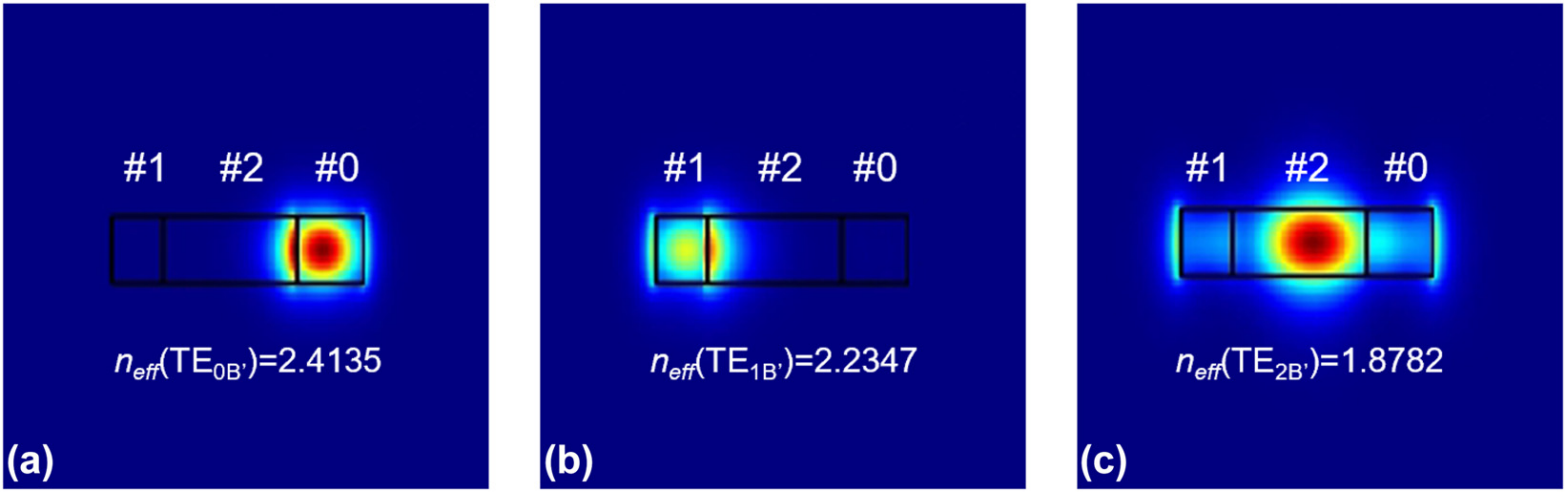
Calculated redistributed mode fields of the supermodes TE_0B′_ (a), TE_1B′_ (b) and TE_2B′_ (c) with the corresponding peaks.

Here the SWG structure is analyzed according to the effective medium theory [49]. Accordingly, the SWG structure is equivalent as a uniform medium with a refractive index given by

(2) $$n^{2}=n_{\text{Si}}^{2}+n_{SiO_{2}}^{2}\left(1-f\right),$$

where *n*_Si_ and *n*_SiO2_ are the refractive indices of the silicon core and the SiO_2_ cladding, and *f* is the SWG duty-cycle. The period and the duty cycle of the SWG structure are chosen as *Λ* = 210 nm and *f* = 0.5 according to the subwavelength-regime condition and the fabrication process. Notably, the fabrication might not be easy for some silicon photonic foundries with UV lithography due to the small feature size of 105 nm. Alternatively, the fabrication is still feasible for the e-beam lithography and deep-UV lithography, and thus the period is chosen as 105 nm here to be with the desired subwavelength-regime operation. According to the design rules, the widths *w*_0_, *w*_1_ and *w*_2_ is chosen as 0.4, 0.3, and 0.8 μm respectively as an example. The redistributed mode fields of the supermodes TE_0B′_, TE_1B′_ and TE_2B′_ are calculated, as shown in Figure 4(a)–(c), respectively. It can be seen clearly that these three modes are successfully localized at regions #0, #1 and #2 in the MBW as desired.

**Figure 5:**
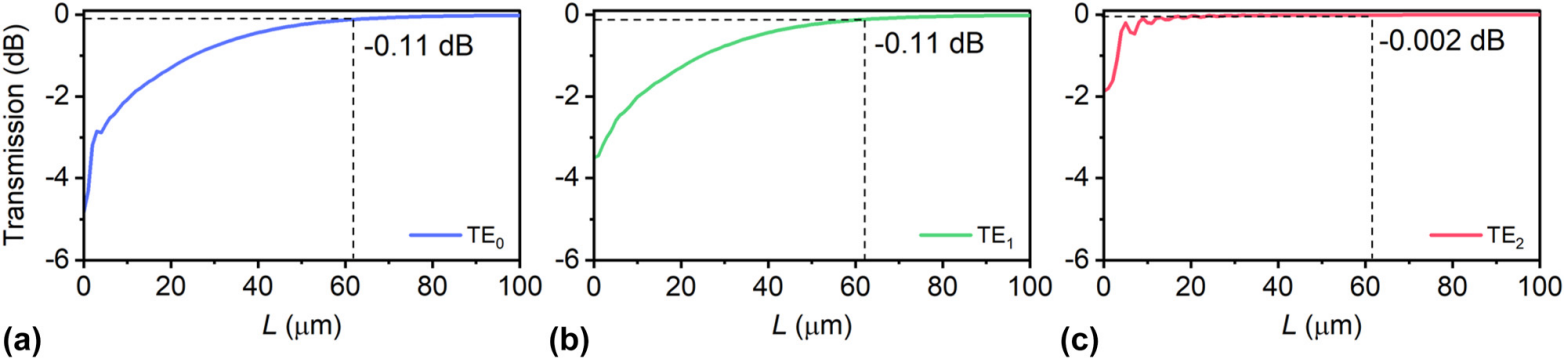
Simulated transmissions of the launched TE_0_ (a), TE_1_ (b) and TE_2_ (c) modes propagating in an adiabatic SWG-assisted taper as the taper length *L* varies.

As shown in Figure 3(a), an adiabatic SWG-assisted taper is introduced to efficiently convert the launched modes to the redistributed supermodes. According to the simulated transmissions of the launched TE_0_, TE_1_ and TE_2_ modes shown in Figure 5(a)–(c), we choose the taper length *L* as 63 μm to ensure the adiabatic mode conversion as well as minimize the footprint.

**Figure 6:**
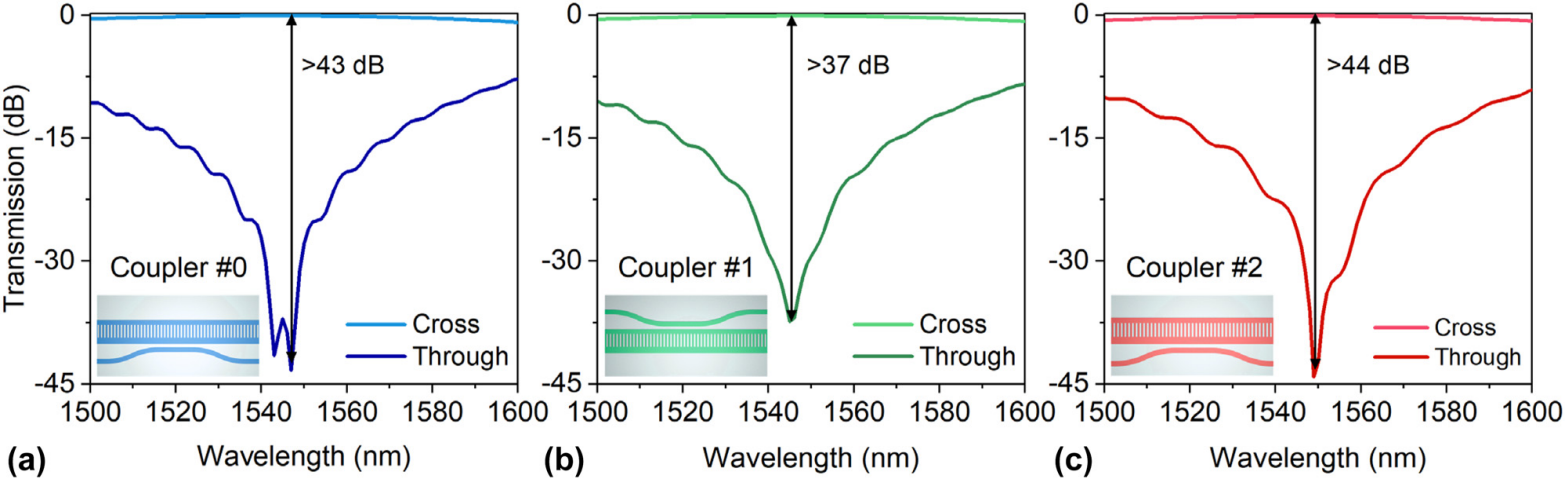
Simulated transmissions at the cross port and the through port of the mode-selective couplers #0 (a), #1 (b) and #2 (c) with optimized parameters.

Then the coupling region should be designed carefully so that each mode channel can be added/dropped with high mode selectivity. The width of the access waveguide in the coupling region is chosen optimally to have efficient evanescent coupling for the desired mode-channel in the MBW according to the phase matching condition, so that the fundamental mode in the access waveguide can be matched (i.e., *δ* ≈ 0) to the corresponding supermode in the MBW. The waveguide parameters are optimized carefully by monitoring the transmissions of the cross port and the through port of these couplers. Here a three-dimensional finite-difference time-domain (3D-FDTD) method was used for simulating the light propagation. For couplers #1 and #2 used for the TE_1_- and TE_2_-supermodes, the access waveguides are placed at the sides of regions #1 and #0, respectively, as shown in Figure 3(a). Figure 6(a)–(c) show the calculated transmissions at the cross port and the through port of the mode-selective couplers #0, #1 and #2 with optimized parameters. The corresponding access waveguides are optimized to be with the core width of 474, 397, and 300 nm, respectively. Here the length of the coupling region is defined as *L*_c_ = *NΛ*, where *N* is the number of the grating period. Accordingly, the optimized coupling lengths *L*_c_ are chosen as 18.9, 8.40 and 3.36 μm for the TE_0B′_, TE_1B′_ and TE_2B′_ supermodes, respectively, when choosing the gap widths as 160, 160 and 200 nm. The length of the S-bend *L*_s_ is set as 10 μm and the gap *D* between the S-bend and the MBW is chosen as 1 μm for all the mode channels. All the key parameters are summarized in Table 1. From the optimized result shown in Figure 6, it can be seen that the extinction ratio between the cross port and the through port are higher than 43 dB, 37 dB and 44 dB for the TE_0_, TE_1_ and TE_2_ mode-channels around the central wavelength. It is also noticed that the extinction ratios are higher than 20 dB over a wavelength range of 28 nm for all three modes. The designed couplers also exhibit low excess losses of 0.06–0.6 dB in the bandwidth of 1500–1600 nm.

**Table 1:** Structure parameters.

Channel	*w*_g_ (nm)	*w*_a_ (nm)	*L*_c_ (μm)
TE_0_	160	474	18.9 (*N* = 90)
TE_1_	160	397	8.40 (*N* = 40)
TE_2_	200	300	3.36 (*N* = 16)

**Figure 7:**
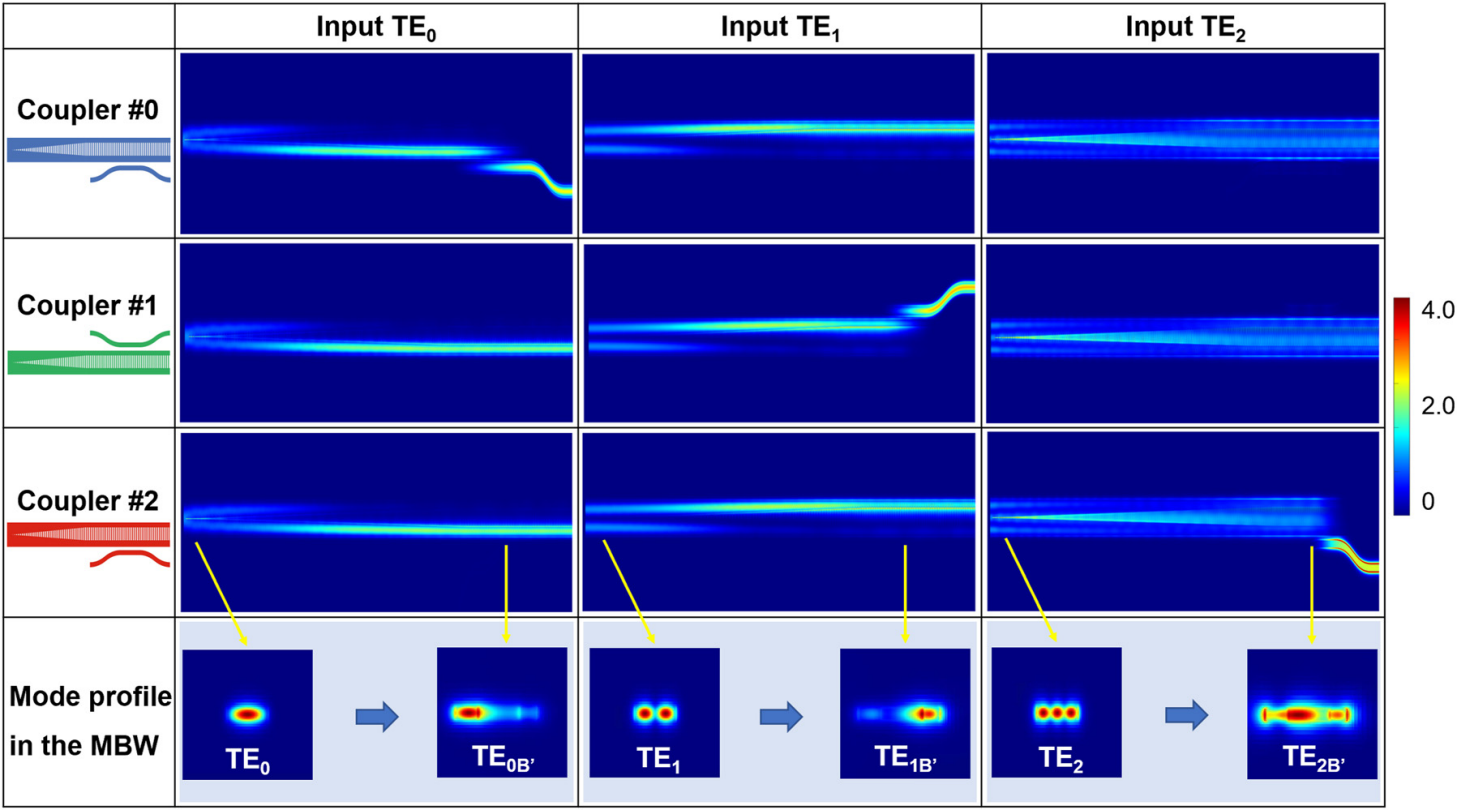
Simulated light propagation in the whole mode-selective coupler structure when the TE_*i*_ (*i* = 1, 2, and 3) mode-channel in the MBW is launched.

Figure 7 shows the simulated light propagation in the designed couplers for the TE_0_, TE_1_ and TE_2_ mode-channels when operating at 1550 nm, including the mode evolution region and the mode coupling region. As it can be seen, when the TE_*i*_ (*i* = 1, 2, and 3) mode is launched at the input end of the mode evolution region, the mode profile is redistributed and converted to the TE_*i*B′_ (*i* = 1, 2, and 3) supermode respectively localized at regions #0, #1, and #2 of the MBW. When the TE_*i*B′_ (*i* = 1, 2, and 3) supermode is launched at the input end of the corresponding coupler, it is finally coupled to the access waveguide with high efficiency. Meanwhile, the other TE_*j*_ modes (*j* ≠ *i*) pass through with negligible ELs. There is no notable crosstalk observed, indicating that these designed couplers work well with all the mode-channels. When using these mode-selective coupler, the undesired mode coupling is suppressed significantly. Therefore, any mode channels can be selectively added to or dropped from the MBW by placing an optimized access waveguide at the particular side of the MBW.

**Figure 8:**
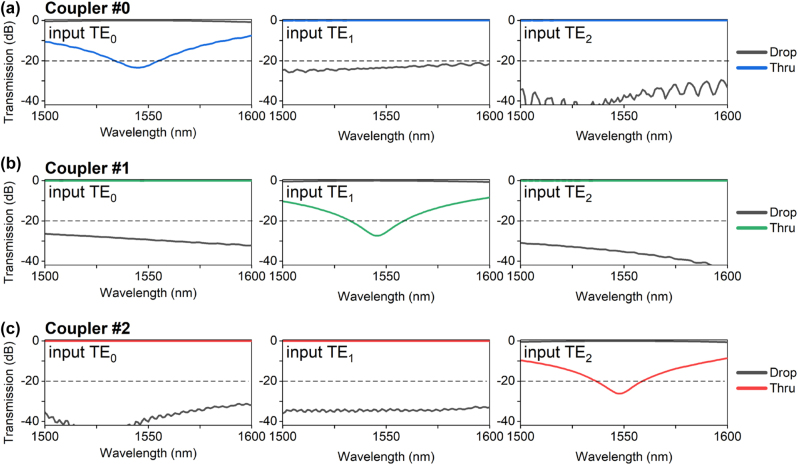
Simulated transmissions in the whole mode-selective coupler structure when the TE_*i*_ (*i* = 1, 2, and 3) mode-channel is launched in the MBW.

Figure 8(a)–(c) show the simulated transmissions for the TE_0_, TE_1_ and TE_2_ mode-channels in the whole mode-selective coupler structures (including the mode evolution region and the mode coupling region), respectively. It shows that all the mode-channels have ELs of <0.3 dB over the wavelength range of 70 nm, which is reasonable regarding the wavelength-dependence of directional couplers. It is noteworthy that the inter-mode crosstalks are low for couplers #0, #1, and #2 when the TE_*j*_ (*j* ≠ *i*) mode is launched, indicating that the present mode manipulation scheme works well by utilizing the mode field redistribution to suppress the coupling ratio of the undesired mode. For coupler #0, the inter-mode crosstalk at the central wavelength is <−23.7 dB and −37.1 dB when TE_1_ and TE_2_ mode is input from the MBW, respectively. For coupler #1, it has a lower inter-mode crosstalk of −26.5 ∼ −32.1 dB (TE_0_ input) and −31.1 ∼ −42.8 dB (TE_2_ input). For coupler #2, the inter-mode crosstalk is lower than −31.3 dB in the wavelength range of 1500–1600 nm for all the input modes. The crosstalks between the drop- and through-ports for coupler #*i* when TE_*i*_ mode is launched are <−23.4 dB, −27.4 dB and −26.2 dB at the central wavelength. Note that the crosstalk here is slightly higher than that shown in Figure 6 (which is for the mode coupling region only), which means that the mode evolution region might introduce some crosstalks as well. Furthermore, the supermodes cannot be localized perfectly and some crosstalk is also introduced.

The coupling coefficient *κ* and the ratio *δ*/*κ* for each guided-mode of a traditional ADC (shown in Figure 2(a)) and the proposed mode-selective coupler are calculated by Eq. (3) according to the couple mode theory [48], as shown in Tables 2 and 3, respectively. The widths of the bus waveguide are both chosen as 1.5 μm. The core width *w*_A_ of the access waveguide for the traditional ADC is chosen as 1.5, 0.74 and 0.48 μm for coupler #0, #1 and #2, as mentioned in Figure 2(b), while the access waveguide width *w*_a_ of the proposed mode-selective coupler is chosen according to Table 1. The gap width of the traditional ADC is chosen to be as same as the proposed coupler. It can be seen that the coupling coefficient of the desired mode (i.e., the TE_*i*_ mode for coupler #*i*) is maximized and that of the undesired modes (i.e., the TE_*j*_ mode for coupler #*i*, *j* ≠ *i*) are minimized in this work, compared with traditional ADCs. One might notice that the TE_0_ mode in coupler #2 has the largest coupling coefficient *κ* (higher than the desired TE_2_ mode). On the other hand, one should also notice that the desired TE_2_ mode in coupler #2 has a much lower ratio *δ*/*κ* than the TE_0_ mode, which is the determinant key to achieve the strongest evanescent coupling for the TE_2_ mode (other than the TE_0_ mode) as desired.

(3) (3) $$\kappa_{pq}=\frac{\omega \varepsilon_{0}\int_{-\infty }^{\infty }\int_{-\infty }^{\infty }\left(N^{2}-N_{q}^{2}\right)E_{p}^{*}\cdot E_{q}dxdy}{\int_{-\infty }^{\infty }\int_{-\infty }^{\infty }u_{z}\cdot \left(E_{p}^{*}\times H_{p}+E_{p}\times H_{p}^{*}\right)dxdy},$$

**Table 2:** Calculated coupling coefficient *κ* (×10^3^ m^−1^).

	Traditional ADC			This work		
	TE_0_	TE_1_	TE_2_	TE_0_	TE_1_	TE_2_
Coupler #0	1.725	7.706	25.92	66.92	7.584	28.81
Coupler #1	4.535	7.671	9.047	2.383	94.66	41.14
Coupler #2	9.684	15.91	18.12	314.3	28.87	97.59

**Table 3:** Calculated ratio *δ*/*κ*.

	Traditional ADC			This work		
	TE_0_	TE_1_	TE_2_	TE_0_	TE_1_	TE_2_
Coupler #0	4.053/1.725	4.053/7.706	4.053/25.92	4.053/66.92	755.2/7.584	2178/28.81
Coupler #1	564.6/4.535	4.053/7.671	1020/9.047	760.1/2.383	4.053/94.66	1438/41.14
Coupler #2	1575/9.684	1004/15.91	4.053/18.12	2353/314.3	1614/28.87	4.053/97.59

## 3 Fabrication and measurement

**Figure 9:**
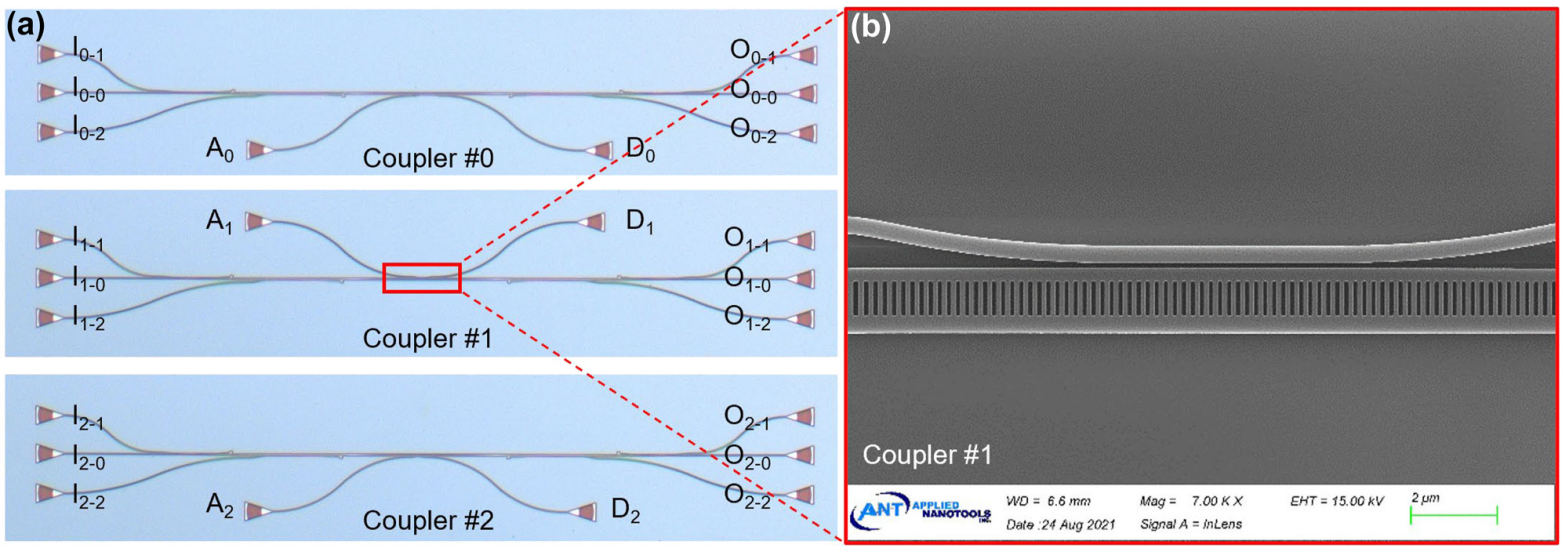
Images of the fabricated device. (a) Microscope images of the fabricated PIC for coupler #0, #1 and #2, including a pair of mode (de)multiplexers based on dual-core adiabatic tapers. (b) Scanning electron microscope (SEM) image of the coupling region.

As a proof of concept, the designed mode-selective add-drop couplers with three mode-channels utilizing the proposed mode-selective manipulation scheme were then fabricated at Applied Nanotools on an SOI wafer with a 220-nm-thick top-silicon layer and a 2-μm-thick buried oxide layer. The E-beam lithography (EBL) was used to pattern, and an inductively coupled plasma (ICP) dry-etching process was applied for the silicon core etching. By utilizing the plasma enhanced chemical vapor deposition (PECVD) process, a 2-μm-thick SiO_2_ thin film was deposited on top of the silicon core layer as the cladding. Figure 9(a) shows the microscope image of the fabricated photonic integrated circuit (PIC) for coupler #0, #1 and #2. In order to characterize the fabricated PIC, a pair of three-channel mode (de)multiplexer based on dual-core adiabatic tapers [38] were used for (de)multiplexing the TE_0_, TE_1_ and TE_2_ modes. Grating couplers were applied to realize efficient fiber-chip coupling. The zoom-in scanning electron microscope (SEM) image of the SWG coupling region is shown in Figure 9(b), indicating that the PIC is fabricated well. Notably, some fabrication imperfectness might exist, mainly including the angled sidewalls (for which the measured angle is ∼88° in this work) due to the imperfect dry-etching process and the SiO_2_ voids in the SWG structure caused by the PECVD process. The sidewall angle and the SiO_2_ voids introduce some change for the effective indices of the redistributed supermodes, indicating that the coupling of the desired mode becomes imperfect due to the increased phase mismatch *δ*. On the other hand, the undesired mode coupling is still kept to be sufficiently low because the phase mismatching is still high.

**Figure 10:**
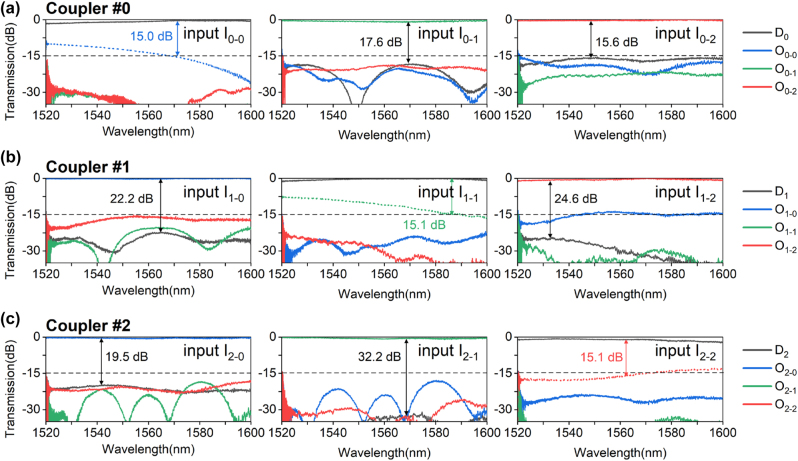
Normalized transmission spectra at drop-port D_*i*_ (*i* = 0, 1, and 2) and output-port O_*i*−*j*_ (*i*, *j* = 0, 1, and 2) for coupler #0 (a), coupler #1 (b) and coupler #2 (c) as light is launched at each input port I_*i*−*j*_ (*i*, *j* = 0, 1, and 2).

In order to characterize the performance of the fabricated PICs, a broadband amplified spontaneous emission (ASE) light source was used and light is coupled into any one of the input ports I_*i*−*j*_ (*i*, *j* = 0, 1, and 2) through grating couplers, so that the TE_0_, TE_1_ and TE_2_ modes can be excited for launching selectively. An optical spectrum analyzer (OSA) was used to record the transmission spectrum of drop port D_*i*_ (*i* = 0, 1, and 2) and output port O_*i*−*j*_ (*i*, *j* = 0, 1, and 2). The fiber angle used for measurements is 13°. Figure 10(a)–(c) show the measured transmissions at drop port D_*i*_ and output port O_*i*−*j*_ when light is launched from ports I_*i*−*j*_, respectively. Here a straight singlemode waveguide fabricated on the same chip was used for the normalization of the transmissions. For the PIC consisting of a pair of mode (de)multiplexers and the present mode-selective add-drop coupler assisted with modal-field redistribution, the ELs for the drop ports are 0.1–1.9 dB in the wavelength range of 70 nm. Moreover, the other mode channels passed through the device with very low ELs. The inter-mode crosstalk at the drop port D_*i*_ of coupler #*i* for light launched at the ports I_*i*−*j*_ (*j* ≠ *i*) is <−15.6, −22.2 and −19.5 dB in the wavelength range of 1525–1600 nm for the TE_0_, TE_1_ and TE_2_ mode-channels, respectively. One might notice that there is some crosstalk (noise) around 1520 nm, which is mainly due to the bandwidth limitation of the ASE source and the grating couplers.

**Figure 11:**
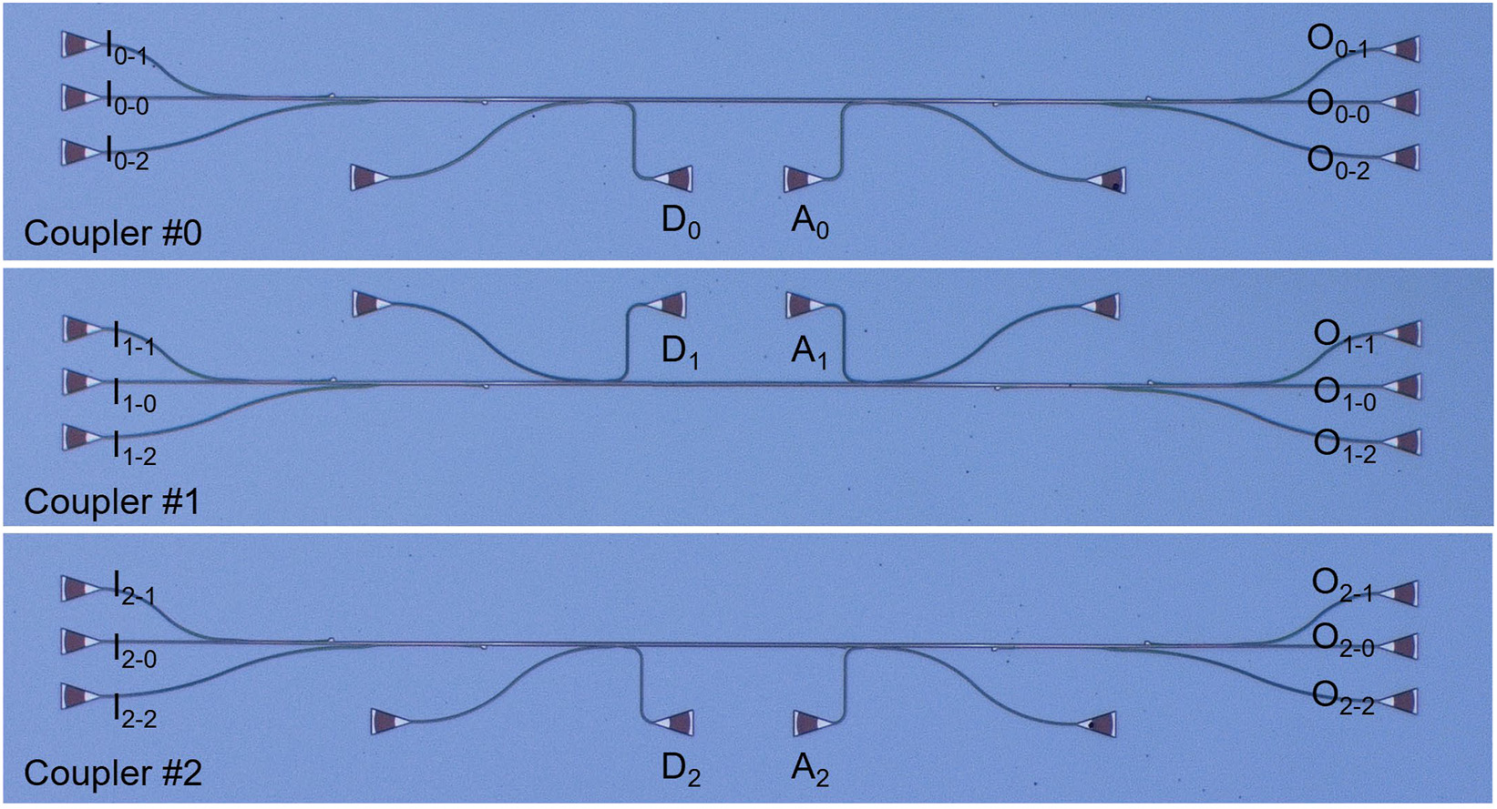
Microscope images of the improved PIC for the TE_0_, TE_1_ and TE_2_ mode-selective coupler #0, #1 and #2 with an additional coupler in cascade.

**Figure 12:**
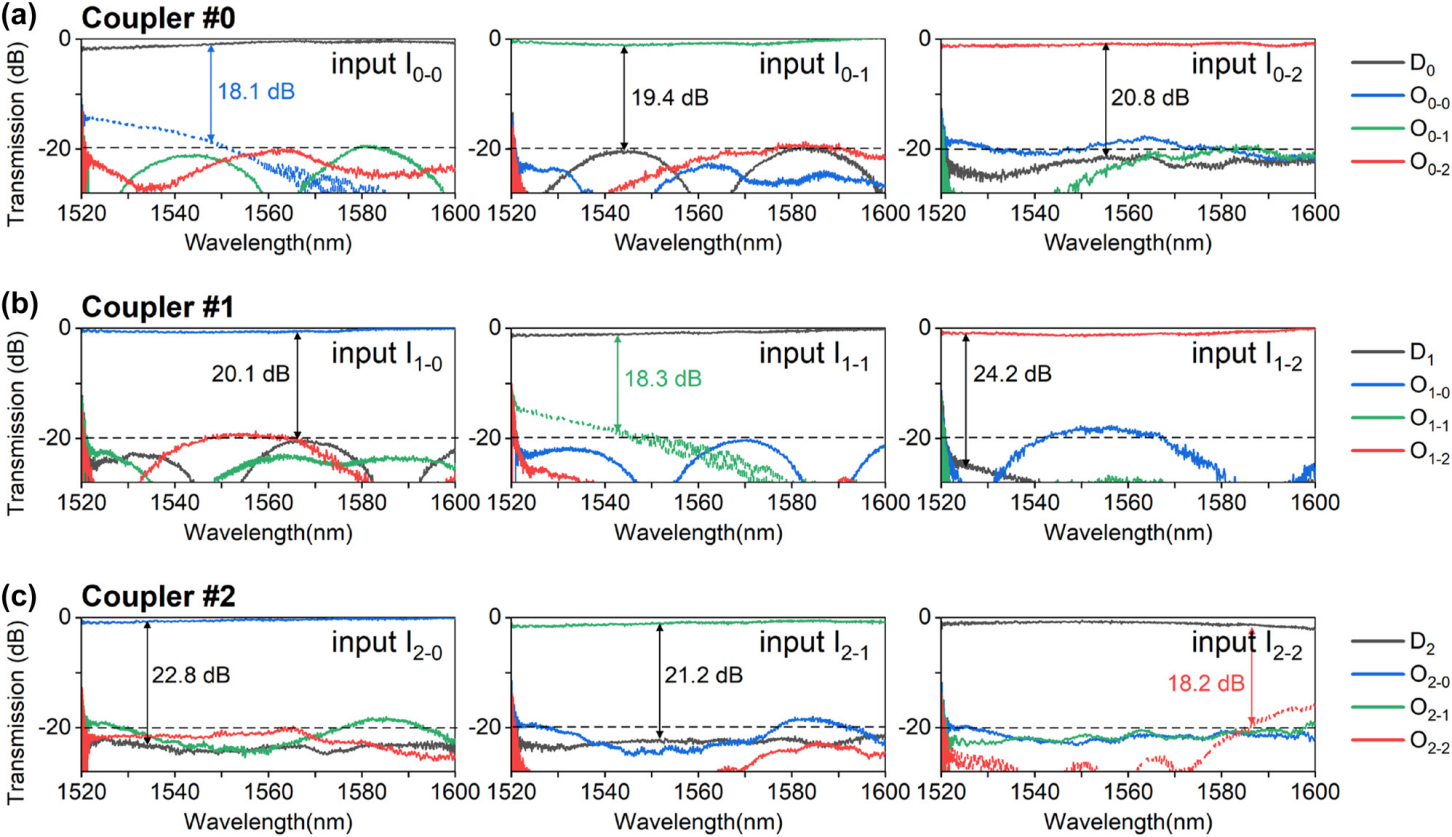
Normalized transmission spectra at drop port D_*i*_ (*i* = 0, 1, and 2) and output port O_*i*−*j*_ (*i*, *j* = 0, 1, and 2) when the TE_0_ (a), TE_1_ (b) and TE_2_ (c) modes are launched respectively at input port I_*i*−*j*_ (i, j = 0, 1, and 2) for the improved PIC with an additional coupler in cascade.

We also observed that there is some residual power at port O_*i*−*i*_ when the TE_*i*_ mode channel is launched (see the dashed lines in Figure 10(a)–(c)). This is because the coupling from the bus waveguide to the drop port is incomplete due to the fabrication imperfectness possibly caused by the angled sidewalls and the SiO_2_ voids mentioned above, leading to some crosstalk increment for the mode channel to be added/dopped. This can be further improved by introducing an additional coupler in cascade to filter the residual power of the TE_*i*_ mode-channel in the MBW, as shown in Figure 11, in which way the transmission at port O_*i*−*i*_ can be suppressed greatly. Figure 12(a)–(c) show the measured transmissions at the drop and output ports. It can be seen that the residual power for the TE_*i*_ mode at port O_*i*−*i*_ are significantly reduced to be −18 ∼ −30 dB in the wavelength range of ∼60 nm. Meanwhile, the inter-mode crosstalks for coupler #*i* at the drop port D_*i*_ when light is launched at the ports I_*i*−*j*_ (*j* ≠ *i*) are <−19.4 dB in the wavelength range of 1525–1600 nm. Such mode manipulation scheme is possible to work with other multimode devices with improved performances in the future.

## 4 Conclusions

In summary, we have proposed and demonstrated a novel mode-selective manipulation scheme based on the modal-field redistribution assisted with SWG structure. To the best of our knowledge, it is the first time to propose such a concept for manipulating the evanescent coupling in multimode photonic systems. Particularly, we have focused on manipulating the coupling coefficient *κ* as well as the ratio *δ/κ* in mode-selective coupling systems, which is totally different from the design of traditional ADCs. By using the SWG structure to engineer the refractive-index and the mode profile, the launched modes are redistributed to be localized in different local regions. Consequently, the coupling coefficient of the desired mode-channel is enhanced while that of the undesired mode are highly suppressed, which results in low inter-mode crosstalk. With the help of such mode manipulation scheme, all the mode channels (particularly including the fundamental mode) in the bus waveguide can be added/dropped independently and freely. As a proof of concept, a three-channel mode-selective add-drop coupler utilized the proposed mode manipulation scheme is fabricated and demonstrated experimentally. The designed coupler shows low ELs of <0.3 dB over a wavelength range of 70 nm for all three mode channels. The inter-mode crosstalks of the coupler #0, #1 and #2 for the TE_0_, TE_1_ and TE_2_ mode-channels are lower than −23.7 dB (TE_1_ input), −26.5 dB (TE_0_ input) and −31.1 dB (TE_0_ input), respectively. For the fabricated devices, the ELs for the drop ports are 0.1–1.9 dB in the wavelength range of 70 nm. Furthermore, an additional coupler in cascade has been introduced to filter the residual power of the TE_*i*_ mode-channel in the MBW and as a result the transmission at port O_*i*−*i*_ is suppressed greatly to be as low as −18 ∼ −30 dB in the wavelength range of ∼60 nm. Meanwhile, the inter-mode crosstalks are <−19.4 dB in the wavelength range of 1525–1600 nm. The proposed concept of manipulating the evanescent coupling of the mode-channels in the multimode bus waveguide is promising for the realization of multimode silicon photonic circuits for flexible mode-selective manipulation in the future.
